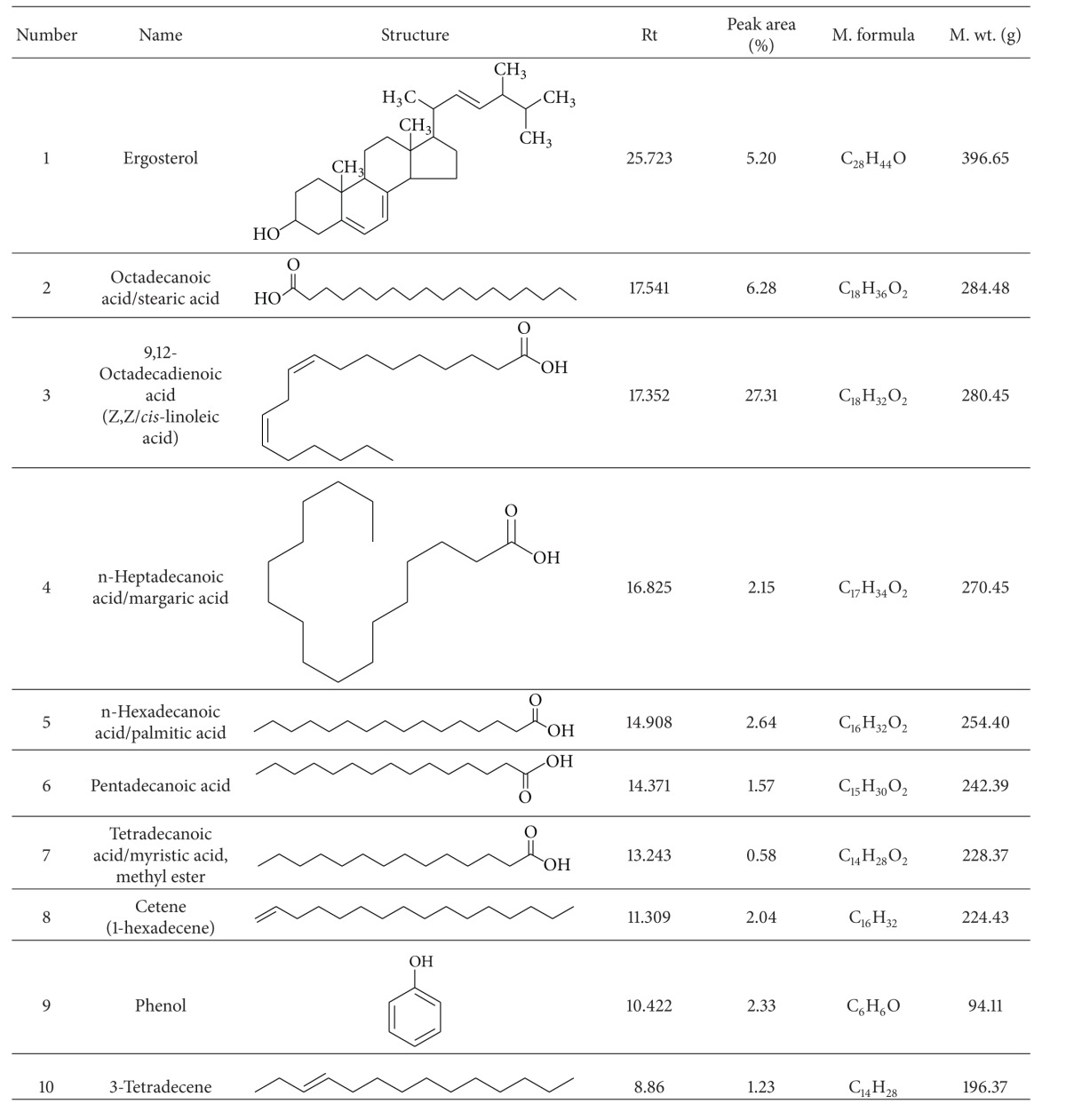# Inhibitory Effect on *In Vitro* LDL Oxidation and HMG Co-A Reductase Activity of the Liquid-Liquid Partitioned Fractions of *Hericium erinaceus* (Bull.) Persoon (Lion's Mane Mushroom)

**DOI:** 10.1155/2014/828149

**Published:** 2014-05-13

**Authors:** Mohammad Azizur Rahman, Noorlidah Abdullah, Norhaniza Aminudin

**Affiliations:** ^1^Mushroom Research Centre, Institute of Biological Sciences, Faculty of Science, University of Malaya, 50603 Kuala Lumpur, Malaysia; ^2^Department of Biochemistry and Molecular Biology, Jahangirnagar University, Savar, Dhaka 1340, Bangladesh

## Abstract

Oxidation of low-density lipoprotein (LDL) has been strongly suggested as the key factor in the pathogenesis of atherosclerosis. Mushrooms have been implicated in having preventive effects against chronic diseases due especially to their antioxidant properties. In this study, *in vitro* inhibitory effect of *Hericium erinaceus* on LDL oxidation and the activity of the cholesterol biosynthetic key enzyme, 3-hydroxy-3-methyl glutaryl coenzyme A (HMG Co-A) reductase, was evaluated using five liquid-liquid solvent fractions consisting of methanol : dichloromethane (M : DCM), hexane (HEX), dichloromethane (DCM), ethyl acetate (EA), and aqueous residue (AQ). The hexane fraction showed the highest inhibition of oxidation of human LDL as reflected by the increased lag time (100 mins) for the formation of conjugated diene (CD) at 1 µg/mL and decreased production (68.28%, IC_50_ 0.73 mg/mL) of thiobarbituric acid reactive substances (TBARS) at 1 mg/mL. It also mostly inhibited (59.91%) the activity of the HMG Co-A reductase at 10 mg/mL. The GC-MS profiling of the hexane fraction identified the presence of myconutrients: *inter alia*, ergosterol and linoleic acid. Thus, hexane fraction of *Hericium erinaceus* was found to be the most potent *in vitro* inhibitor of both LDL oxidation and HMG Co-A reductase activity having therapeutic potential for the prevention of oxidative stress-mediated vascular diseases.

## 1. Introduction

Atherosclerosis, a complex pathophysiological process, is the cause for more than half of all the deaths in the developed world [1]. Oxidative stress-induced modification of plasma lipoproteins, especially low-density lipoprotein (LDL) and elevated cholesterol level, has been highly implicated among the multiple causative factors of atherosclerosis [2–5]. In fact, numerous oxidation-prone free radicals such as superoxide, peroxide, nitrogen oxide, and nitric oxide are abundantly produced and removed very often in normal physiological conditions [6]. Imbalance between their production and removal resulting in excessive free radicals afflicts the normalcy that turn into pathophysiological consequences [7]. Atherosclerosis stands among the most common of such concerns that ultimately leads to cardiovascular diseases (CVD) and strokes [8–10]. Factors governing oxidation of LDL set the ground for the vicious cycle of atherosclerosis [5, 11, 12]. Oxidized LDL (ox-LDL) is readily internalized by macrophages. Atherosclerosis recruits macrophages into arterial wall, wherein they turn into lipid-laden foam cells. Foam cells, later on, perpetuates inflammatory responses that cause further recruitments of monocytes and macrophages into the sub endothelial spaces, leading to endothelial injury and dysfunctions [5, 12, 13]. Strategies provided with maintenance of reduced oxidative stress and improved antioxidant defense had generated both “positive” and “no response” effects in case of atherosclerosis and CVD. The reason behind this mixed outcome had been attributed to the dosage of the antioxidants used, the distinction between* in vitro* and* in vivo* experimental models, and occurrence of multimodal oxidative stress that goes beyond the defense capacity of any specific antioxidant [14]. Thus, development of antiatherosclerotic potential of antioxidants warrants appropriate therapeutic approaches.

Natural foods spur high in combating atherosclerosis through their effect on preventing LDL oxidation [15], lowering plasma cholesterol, or inhibiting the biosynthesis of cholesterol [16]. Edible fungi such as mushrooms, being abound in myconutrients, beacon excellent in this context [17–19]. Mushrooms have been highly regarded for possessing natural free radical scavengers of various sorts including polysaccharides (e.g., *β*-D glucan), polyphenols (e.g., phenolic acids, flavonoids), vitamins (e.g., tocopherol, ascorbic acid, niacin), ergosterol, and carotenoids. Bioactive compounds present in mushrooms confer them to be pluripotential agent for antioxidant [20–22], antitumor/anticancer [23, 24], antimicrobial [25, 26], immunomodulatory [23, 27], antiatherogenic [28–31], and hypoglycemic actions [32, 33]. Mushrooms' hypocholesterolemic effect has been attributed to their inhibitory effect on cholesterol biosynthesis [34] and absorption [35, 36] and stimulatory effect on fecal excretion [37]. The dietary fibers present in mushrooms further reinforce their suitability for being hypolipidemic agents [38, 39].

Bioactive components present in mushrooms warrant appropriate separation, recovery, and purification processes for obtaining maximum output from their usage. Liquid-liquid partitioning, also called solvent partitioning, is a rapid, inexpensive, and steady-state procedure for extracting biocomponents [40]. Through this approach, compounds are separated based on their relative solubility in two different immiscible solvents, most commonly water and an organic solvent [41]. The organic solvent to be used is determined depending on the chemical and physical properties of the biocomponents to be separated. Also, the density difference between water and the solvent should be high; water-solvent interfacial tension should allow their contact well; solvent must not react irreversibly with the components and the recovery rate of the components should be high after partitioning [40]. Both polar (e.g., ethanol, methanol) and nonpolar solvents (e.g., dichloromethane, hexane) or their mixture (e.g., methanol : dichloromethane) is used depending on the bioactive components of interest. When left undisturbed, the solvents form two distinct layers, in each fraction containing the component of respective solubility. The soluble compound is separated from the insoluble one by rotary evaporation or by any other downstream processing.

*Hericium erinaceus, *belonging to the division Basidiomycota and class Agaricomycetes, is both an edible and medicinal mushroom. It is popular across the continents in delicacy and replaces pork or lamb in Chinese vegetarian cuisine. Its effect on cognitive improvement [42], stimulating nerve growth factors [43] and nerve cells [44], ameliorating effect, and hypoglycemic effect [45] have been reported. However, there is still paucity of information indicating its role in atherosclerosis. Thus, the present study was designed to prepare liquid-liquid partitioned fractions of* H. erinaceus* and elucidate their antiatherosclerotic potential through evaluation of* in vitro* inhibitory effect on LDL oxidation and HMG Co-A reductase activity followed by the identification of the bioactive components present in the most potent fraction.

## 2. Materials and Methods

### 2.1. Solvent Partitioning and Preparation of Liquid-Liquid Fractions

The modified method of Mayakrishnan et al. [46] was applied for solvent partitioning and fractionation of* Hericium erinaceus*. Purchased from the local farm, the mushroom fruit body was sliced, sun-dried, and ground to powder. Two hundred grams of powder was extracted with 4 L of methanol : dichloromethane (2 : 1) in conical flasks at room temperature with occasional stirring and shaking for 3 days followed by filtration through Whatman number 1 filter paper. The extraction was repeated twice and the total organic solution, collected from each step of extraction, was evaporated using a rotary evaporator (Büchi Rotavapor R-114, Switzerland) that yielded the crude (M : DCM) extract. The dried, crude extract was dissolved in 90% aqueous methanol and partitioned with hexane (3 × 100 mls). The upper hexane layer was separated using a separating funnel and hexane rota-evaporated. The bottom aqueous methanolic layer left was rota-evaporated which yielded a semisolid fraction. Redissolving of the semisolid fraction in distilled water (100 mL) was followed by successive partitioning with dichloromethane (DCM, 3 × 100 mls). The bottom-layered DCM fraction was collected and DCM rota-evaporated leaving the lowered aqueous fraction to be repartitioned with ethyl acetate (EA, 3 × 100 mls). The upper EA layer was rota-evaporated and the lowered aqueous part freeze-dried to gain the respective fraction.

### 2.2. Sources of the Chemicals

The chemicals used in the present study were of analytical grade and purchased from Sigma-Aldrich (USA).

### 2.3. FeSO_4_-Induced LDL Oxidation and* H. erinaceus* Fractions-Mediated Inhibition

To determine the effect of the fractions on the inhibition of the oxidation of human low-density lipoprotein (LDL), the method of Ahmadvand et al. [47] was used with some modifications. Two aspects of LDL oxidation were measured: conjugated diene (CD) and thiobarbituric acid reactive substances (TBARS).

#### 2.3.1. Continuous Monitoring of the Formation of CD

For CD measurement, the concentration of LDL was adjusted to 150 *μ*g/mL and the reaction volume totaled to 200 *μ*L with 0.1 M phosphate buffer, pH 7.4. The oxidative stress to LDL molecules was exerted through freshly prepared FeSO_4_ solution of varying concentrations (1, 5, 10, 20, 25, and 50 *μ*g/mL) at room temperature. The kinetics of LDL oxidation and the oxidation-withstanding effect of the extracts were studied at 234 nm at 20-minute intervals for a period of 3 h. As for blank, FeSO_4_ in ultrapure water only, at pH 7.4, was used. A tangent drawn from the oxidation profile of LDL to the slope of the propagation phase and extrapolation into the intercept revealed the lag phase, the antioxidant-shielded phase of the contour LDL oxidation.* Hericium erinaceus* fractions-mediated protection period of the LDL oxidation was dubbed as the “lengthened lag time of CD formation” and was measured until the amount of the CD began to increase.

#### 2.3.2. Assay of the Formation of Thiobarbituric Acid Reactant Substances (TBARS)

The end products of LDL peroxidation, TBARS, were measured as per the modified method of Buege and Aust [48]. Human LDL (9 *μ*L) was mixed with 191 *μ*L of 10 mM ferrous sulphate, the oxidizing agent for the LDL. The oxidized LDL molecules were subjected to 100 *μ*L of 1 *μ*g/mL of extract. To the mixture, 500 *μ*L of 15% trichloroacetic acid (TCA) and 1 mL of 1% thiobarbituric acid (TBA), both freshly prepared, were added and incubated at 100°C for 10 minutes. After cooling down at room temperature, an aliquot (300 *μ*L) was taken to the ELISA reader and the absorbance read at 532 nm. The blank was FeSO_4_ in water, pH 7.4. The percentage inhibition of TBARS formation was calculated using the following equation:

(1) $$\begin{array}{c} \text{Inhibition}\left(\%\right)=\left(A_{0}-A_{s}\right)\times \frac{100}{A_{0}}, \end{array}$$

where *A*_0_ is the absorbance of the control and *A*_s_ is the absorbance of the reaction mixture containing the extract. IC_50_ value (concentration of the extract to produce half maximal inhibition) of the most potent solvent extract was calculated from the graph of the inhibition of TBARS against extract concentration.

### 2.4. Inhibition of the HMG-CoA Reductase Activity

The potentiality of each of the extracts in inhibiting the rate limiting enzyme of cholesterol biosynthesis was measured according to the method of Gholamhoseinian et al. [49] using HMG-CoA reductase assay kit (Sigma, St. Louis, USA; catalog number CS1090) according to the manufacturer's protocol. Pravastatin was used as the standard reference HMG-CoA reductase inhibitor.

The percentage inhibition of the HMG Co-A reductase activity was calculated using the following equation:

(2) $$\begin{array}{c} \text{Inhibition}\left(\%\right)=\left(A_{0}-A_{s}\right)\times \frac{100}{A_{0}}, \end{array}$$

where *A*_0_ is the absorbance of the control and *A*_*s*_ is the absorbance of the reaction mixture containing the extract. IC_50_ value (concentration of the extract to produce half maximal inhibition) of the most potent solvent extract was calculated from the graph of the inhibition of HMG Co-A reductase against extract concentration.

### 2.5. Gas Chromatography-Mass Spectrometry (GC-MS) Analysis of the Hexane Fraction

Gas chromatography directly coupled to a mass spectrometer system was employed for GC-MS analyses of the hexane fraction of* H. erinaceus*. The column used was HP-5 ms silica capillary column (30 m × 250 *μ*m, 0.25 *μ*m film). Other conditions maintained include oven temperature ranging from 70°C (2 min) to 300°C (6 min), finally maintained for 29 min; helium as the carrier gas, flow rate of 1 mL/min; injector temperature 250°C; injection volume 1.5 *μ*L; injection technique splitless; 70 eV ionization energy, electronic ionization (EI) mode; ion source temperature 200°C; scan mass range 50–550 m/z; and the interface line temperature of 300°C. Peak identification was performed comparing with mass spectra of the National Institute of Standards and Technology (NIST 08 and NIST08s) library data.

### 2.6. Statistical Analyses

All the experiments were performed in triplicate and the data presented as mean ± SD. Statistical package SPSS version 16 was used. Analyses were carried out using one-way analysis of variance (ANOVA) and the differences among means were further analyzed by least significance difference (LSD) at 95% level (*P* ≤ 0.05).

## 3. Results

### 3.1. LDL Oxidation Tests

**Figure 1:**
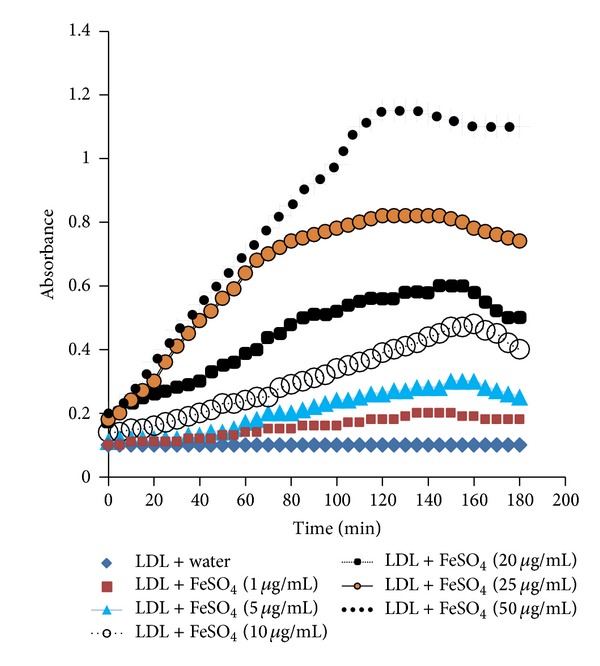
Effect of various concentrations of FeSO_4_ on inducing oxidative modification of human LDL molecules to produce conjugated dienes (CD). The absorbance of formed CD was monitored at 234 nm at every 20 mins for a period of 180 mins.

The oxidative modification of lipid structures and the resulting products are among the key factors that initiate atherosclerotic pathogenesis. The unsaturated portions of lipids especially the double bonds of fatty acids present in lipid molecules are most vulnerable to oxidative attack by free radicals and ions that lead to altered lipid structures resulting in the proatherosclerotic breakdown products [50]. Based on this paradigm, we induced LDL oxidation by Fe^+2^ at low pH and tested the action of each liquid-liquid fraction [47, 50]. All the concentrations (1, 5, 0, 20, 25, and 50 *μ*g/mL) of FeSO_4_ used were capable of inducing oxidation in a dose-dependent manner (Figure 1).

### 3.2. Inhibition of LDL Oxidation by* H. erinaceus* Fractions

**Figure 2:**
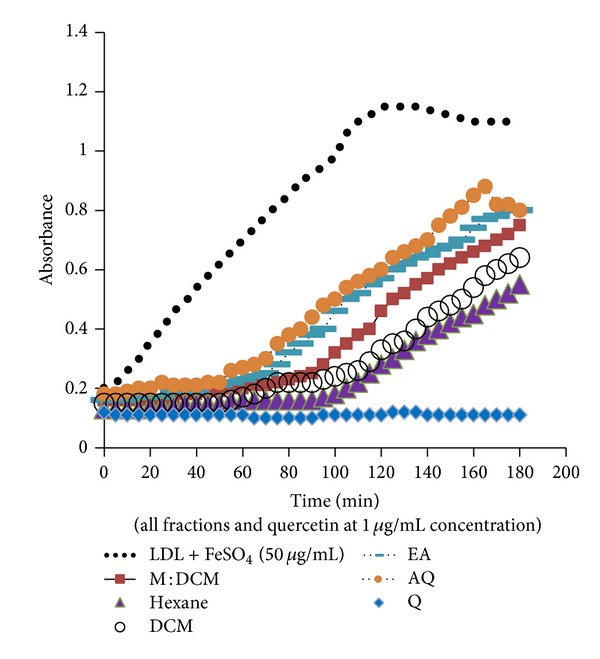
Effect of* H. erinaceus *fractions on inhibiting the oxidation of human LDL (ox-LDL) molecules. The increased lag time of conjugated diene (CD) formation along with decreased absorbance at 234 nm indicates the extent of ox-LDL inhibition by the respective fraction against the FeSO_4_-induced LDL oxidation. Quercetin (Q) was used as the positive control. All the fractions and quercetin were used at 1 *μ*g/mL conc.

**Figure 3:**
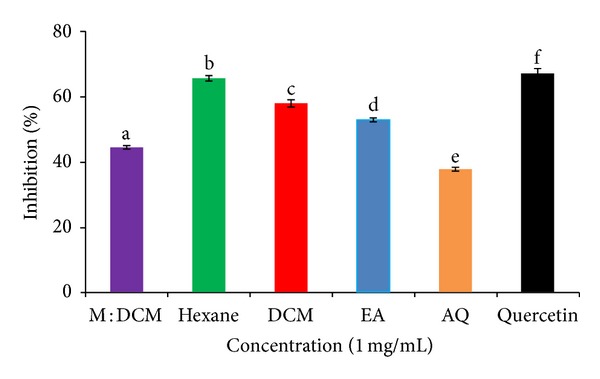
Effect of* H. erinaceus *solvent-solvent fractions on inhibiting the oxidation of human LDL (ox-LDL) molecules. The percentage of inhibition of TBARS production at 532 nm indicates the inhibitory extent of ox-LDL production for the respective fraction. Quercetin (Q) was used as the positive control. All the fractions and quercetin were used at 1 mg/mL conc. Data are presented as mean ± SD of triplicate determinations. Mean values with different lower case superscripts (a–f) represent statistically significant difference at 95% level (*P* ≤ 0.05) with* post hoc* least significance difference (LSD) test.

As LDL oxidation is one of the preliminary steps of atherosclerosis, it is of paramount importance to search for agents capable of withstanding oxidation of LDL with a view to combating atherosclerosis. In this connection, we evaluated the potency of* H. erinaceus* solvent partitioned fractions in inhibiting* in vitro *oxidation of human LDL and monitored their effect on ox-LDL breakdown products CD (Figure 2) and TBARS (Figure 3).

For CD formation, there has been a consensus that increase in lag time of the CD formation indicates the inhibition of LDL oxidation by the antioxidant compound [5, 12]. In the present study, the hexane fraction lengthened the lag time of CD formation the most (100 mins), followed intricately by that of DCM (95 mins) (Figure 2). The lag time of other fractions was shorter (≤70 mins) indicating that the rate of the formation of CD was rapid as evidenced by higher absorbance (Figure 2).

Hexane fraction also most highly inhibited (68.28%, IC_50_ 0.73 mg/mL) the formation of TBARS as a result of the oxidation of LDL molecules (Figure 3). DCM fraction stood second with 58.32% inhibition (IC_50_ 0.87 mg/mL). The sequence of other fractions in decreasing pattern of inhibition was ethyl acetate (51.41%), methyl : DCM (42.11%), and aqueous (37.02%) (Figure 3).

### 3.3. HMG Co-A Reductase Inhibitory Effect

**Figure 4:**
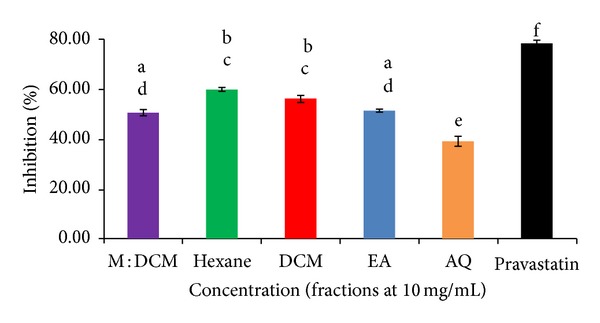
Effect of* H. erinaceus *solvent-solvent fractions on inhibiting the activity of HMG Co-A reductase. All the fractions were 10 mg/mL conc. Pravastatin was used as the positive control directly as per the manufacturer's instructions. Data are presented as mean ± SD of triplicate determinations. Mean values with different lower case superscripts (a–f) represent statistically significant difference at 95% level (*P* ≤ 0.05) with* post hoc* least significance difference (LSD) test.

Elevated plasma cholesterol is one of the most crucial factors of atherosclerosis. Lowering the level of cholesterol reverses or at least retards atherosclerosis. Attempts have been made to monitor the decrease in cholesterol biosynthesis by inhibiting the key enzyme, the 3-hydroxy-3-methyl glutaryl coenzyme A (HMG-CoA) reductase. In this context, we conducted experiments with the solvent partitioned fractions (10 mg/mL conc.) of* H. erinaceus* whether they possess any inhibitory effect upon the HMG-CoA reductase activity* in vitro.* Results obtained demonstrate that all the fractions exerted inhibitory effect on HMG-CoA reductase activity, although varied among themselves in their potency. The hexane fraction showed the highest inhibitory effect of 59.91%, followed by DCM (56.23%) (Figure 4). This trend was in congruence with that of preventing LDL oxidation effects of this fraction. The inhibitory capacity of the other fractions was moderate. Also, inter-fractional variation in reducing the activity of HMG Co-A reductase was not statistically significant. However, the trend of inhibition was similar to that of LDL oxidation prevention by the respective fractions (ethyl acetate 51.41%, M : DCM 50.59%, and aqueous fraction 39.13%) (Figure 4).

### 3.4. Identification of the Chemical Constituents of Hexane Fraction by Gas Chromatography-Mass Spectrmetry (GC-MS)

**Figure 5:**
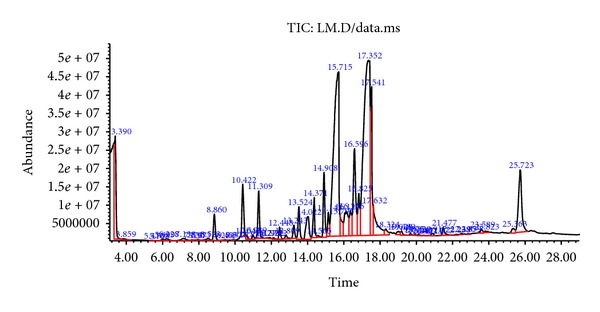
GC-MS chromatogram of the hexane fraction of* Hericium erinaceus*.

Among the five liquid-liquid fractions of* H. erinaceus*, hexane performed the best in withstanding* in vitro* LDL oxidation and HMG Co-A reductase activity. Thus, this fraction was profiled through GC-MS. The GC-MS chromatogram of the hexane fraction is provided in Figure 5 and Table 1. Ten components were characterized based on the peak area percentage, the relative retention time (Rt), and respective quality [51]. Among the most active ones was ergosterol, the major sterol conferring antioxidative effect of the edible mushrooms [52, 53]. Besides, other components belonging to both saturated and unsaturated fatty acids, triterpenes and polysaccharides, were also present. Most of the components identified had preventive role against oxidative stress-induced atherosclerotic pathomechanism [18, 54].

## 4. Discussion

Atherosclerosis is a multifactorial complication leading to cardiovascular diseases and stroke. Elevated level of total cholesterol and LDL-cholesterol stand among the most dreadly risk factors of atherosclerosis. The oxidized form of LDL (ox-LDL) is supposed to set the ground of this pathophysiology. With a view to withstanding atherosclerosis through myconutrient-mediated approach, the present study was designed to* in vitro* evaluate the effect of the solvent partitioned fractions of* H. erinaceus* on ox-LDL, HMG Co-A reductase activity and finally to identify the active components in the most potent fraction by GC-MS analysis.

The* in vitro* oxidation of human LDL molecules was performed using transition metal ion Fe^+2^ [50, 55, 56]. This approach was based on previous research findings that LDL oxidation at cell-free system by redox-active metal ions (Fe, Cu) is physiologically and biochemically similar to that of cellular systems [57–60]. Epidemiologic studies indicate the higher level of iron and copper ions in the arterial walls of the atherosclerotic individuals and thus these redox-active metal ions have been implicated in playing a vital role in oxidizing the native LDL molecule both* in vivo* and* in vitro* [55]. These ions exert peroxidative modification of the polyunsaturated fatty acids (PUFAs) present in the LDL molecule and cause molecular rearrangement, thus forming conjugated dienes (CDs). We used FeSO_4_ at varying doses (1, 10, 20, 25, and 50 *μ*g/mL) and all were capable of the oxidative modification of LDL, as evidenced by the gradual increased absorbance, which is proportional to the formation of CDs. Initially, the endogenous antioxidants present in LDL molecule itself protect LDL by withstanding the oxidative stress and there is suppressed oxidation, also known as the lag time of oxidation. When* H. erinaceus* solvent partitioned fractions were applied, the lag time was lengthened even up to 100 minutes for the hexane and 95 minutes for the DCM fractions. There was a gradual decrease in absorbance of the fraction-treated samples, indicating the decreased oxidation of native LDL molecules and thus reinforcing the anti-LDL-oxidative effect of the fractions.

During the course of LDL oxidation, the lag phase is quickly followed by the propagation phase of rapid LDL oxidation, when lipid peroxides are formed. Then comes the decomposition phase, wherein double bonds are broken and aldehydes, especially malondialdehydes (MDA), are formed. At the present experimental setup, MDA underwent nucleophilic addition reaction with 2-thiobarbituric acid (TBA) and generated “MDA : TBA adduct” dubbed as “thiobarbituric acid reactive substances” (TBARS) [61]. We investigated whether different solvent fractions of* H. erinaceus* could inhibit the formation of TBARS. We found that the hexane fraction had the most potent inhibitory effect on TBARS formation (68.28%, IC_50_ 0.73 mg/mL), followed by DCM (58.32%, IC_50_ 0.87 mg/mL). Ethyl acetate was of moderate activity (51.41%), whereas the effect of methanol : DCM and aqueous fractions were lower (42.11% and 37.02% inhibition, resp.). The high inhibitory effect of the nonpolar hexane fraction might be attributed to its lipophilic myconutrients content.

Antioxidants present in the solvent partitioned fractions of* H. erinaceus* seem pertinent to come into action. Among multiple strategies, the free radical quenching mechanism of the antioxidants holds high suitability in this context. The antioxidant components themselves might become oxidized and thus prevented the LDL molecules from sheer oxidative modifications. Antioxidants may act as electron donors to the free radicals (here, Fe^+2^) to make them stable molecules, thus lessening the oxidative stress towards LDL molecules. Therefore,* H. erinaceus* could be a potent source of compounds preventing LDL oxidation and ox-LDL-mediated atherogenesis.

Hypercholesterolemia is another risk factor for the development of atherosclerosis and attempts have been made for blocking the biosynthesis of cholesterol by inhibiting the activity of the key enzyme of cholesterol biosynthetic pathway, the 3-hydroxy-3-methyl glutaryl coenzyme A (HMG-CoA) reductase [62]. Recently, the HMG Co-A reductase inhibitors have been reported to be having pleiotropic effects beyond cholesterol lowering that involve mainly antiatherogenic performance through antioxidative prevention [63–65]. We tested the effect of* H. erinaceus* solvent fractions on HMG Co-A reductase activity* in vitro,* using pravastatin as the positive control. Here also, the hexane fraction most highly inhibited (59.91%) the activity of HMG Co-A reductase, followed by that of DCM (56.23%). Conceptually,* H. erinaceus* solvent fractions, having anti-ox-LDL potential, were supposed to have inhibitory effect on HMG Co-A reductase activity, and this was found experimentally.

As the hexane fraction showed the best effect for inhibiting oxidization of LDL and HMG Co-A reductase activity, it is very likely that the active mycocomponents might be present here. Thus, we performed GC-MS analysis of the hexane fraction of* H. erinaceus.* Among the most active biocomponents was ergosterol, the major sterol conferring antioxidative effect of the edible mushrooms [52, 53]. Previously, 5,6-dihydroergosterol had been reported to inhibit the production of nitric oxide (NO) and iNOs protein expression and thus aid in preventing inflammatory complications [66]. Hajjaj et al. reported the cholesterol biosynthesis inhibitory effect of another related component, 26-oxygenosterols [67]. The point of inhibition of cholesterol biosynthesis was between lanosterol and lathosterol [67]. Total sterol extracted from mushrooms had been suggested to have ameliorating effect on hypoxia/reoxygenation-induced oxidative stress and inhibitory effect on the formation of reactive oxygen species (ROS) [68].

Octadecanoic acid, another bioactive component present, was found to be associated with lowered LDL-cholesterol in comparison with other saturated fatty acids [69]. Linoleic acid (9,12-octadecadienoic acid), an omega-6 unsaturated fatty acid found in the hexane fraction, has been implicated in having antioxidant effects [70]. Other mycocompounds belonging to saturated and unsaturated fatty acids, triterpenes, and polysaccharides were present. The majority of the components identified had preventive role against oxidative stress-induced atherogenesis [18, 54].

## 5. Conclusion

We have demonstrated that the different solvent partitioned fractions of lion's mane mushroom,* Hericium erinaceus,* possess prowess for preventing LDL oxidation and for withstanding HMG Co-A reductase activity. These august findings suggest strongly that this mushroom species could aid greatly in preventing oxidative stress-induced atherosclerotic pathogenesis and thus is of immense importance for the people suffering from cardiovascular complications and strokes.

## Figures and Tables

**Table 1:** Identification of the active components of the hexane fraction of *H. erinaceus* by GC-MS analysis.